# Supplementary material for: Pyruvate Dehydrogenase Kinase 1 inhibition mediated oxidative phosphorylation enhancement in cartilage promotes osteoarthritis progression
Source: BMC Musculoskelet Disord. 2023 Jul 20;24:597. doi: 10.1186/s12891-023-06585-6 (PMC10357736; doi:10.1186/s12891-023-06585-6)
Supplement: Supplementary file 1 — Additional file 1. [file 12891_2023_6585_MOESM1_ESM.zip › BP.Gsea.1653623667859/GOBP_POST_TRANSLATIONAL_PROTEIN_MODIFICATION.html]

Details for gene set GOBP\_POST\_TRANSLATIONAL\_PROTEIN\_MODIFICATION[GSEA]

|  || Dataset | 55457\_collapsed\_to\_symbols.cls.cls #OA\_versus\_normal.cls.cls #OA\_versus\_normal\_repos |
| Phenotype | cls.cls#OA\_versus\_normal\_repos |
| Upregulated in class | OA |
| GeneSet | GOBP\_POST\_TRANSLATIONAL\_PROTEIN\_MODIFICATION |
| Enrichment Score (ES) | 0.43430668 |
| Normalized Enrichment Score (NES) | 1.5082647 |
| Nominal p-value | 0.028708134 |
| FDR q-value | 0.35910407 |
| FWER p-Value | 1.0 |
Table: GSEA Results Summary

  

Fig 1: Enrichment plot: GOBP\_POST\_TRANSLATIONAL\_PROTEIN\_MODIFICATION      
 Profile of the Running ES Score & Positions of GeneSet Members on the Rank Ordered List

  

| SYMBOL | TITLE | RANK IN GENE LIST | RANK METRIC SCORE | RUNNING ES | CORE ENRICHMENT || 1 | RBX1 | ring-box 1 [Source:HGNC Symbol;Acc:HGNC:9928] | 348 | 0.724 | 0.0700 | Yes |
| 2 | LCMT1 | leucine carboxyl methyltransferase 1 [Source:HGNC Symbol;Acc:HGNC:17557] | 735 | 0.585 | 0.1184 | Yes |
| 3 | ICMT | isoprenylcysteine carboxyl methyltransferase [Source:HGNC Symbol;Acc:HGNC:5350] | 1140 | 0.488 | 0.1524 | Yes |
| 4 | ATG5 | autophagy related 5 [Source:HGNC Symbol;Acc:HGNC:589] | 1142 | 0.488 | 0.2177 | Yes |
| 5 | WIPI2 | "WD repeat domain, phosphoinositide interacting 2 [Source:HGNC Symbol;Acc:HGNC:32225]" | 1612 | 0.411 | 0.2364 | Yes |
| 6 | FN3KRP | fructosamine 3 kinase related protein [Source:HGNC Symbol;Acc:HGNC:25700] | 1664 | 0.404 | 0.2866 | Yes |
| 7 | VIPAS39 | "VPS33B interacting protein, apical-basolateral polarity regulator, spe-39 homolog [Source:HGNC Symbol;Acc:HGNC:20347]" | 1710 | 0.396 | 0.3362 | Yes |
| 8 | UCHL3 | ubiquitin C-terminal hydrolase L3 [Source:HGNC Symbol;Acc:HGNC:12515] | 1844 | 0.379 | 0.3767 | Yes |
| 9 | DSCC1 | DNA replication and sister chromatid cohesion 1 [Source:HGNC Symbol;Acc:HGNC:24453] | 2362 | 0.319 | 0.3793 | Yes |
| 10 | AGBL5 | AGBL carboxypeptidase 5 [Source:HGNC Symbol;Acc:HGNC:26147] | 2613 | 0.290 | 0.3987 | Yes |
| 11 | P3H1 | prolyl 3-hydroxylase 1 [Source:HGNC Symbol;Acc:HGNC:19316] | 2853 | 0.265 | 0.4156 | Yes |
| 12 | RNF7 | ring finger protein 7 [Source:HGNC Symbol;Acc:HGNC:10070] | 3264 | 0.225 | 0.4139 | Yes |
| 13 | PPIB | peptidylprolyl isomerase B [Source:HGNC Symbol;Acc:HGNC:9255] | 3372 | 0.214 | 0.4343 | Yes |
| 14 | COPS5 | COP9 signalosome subunit 5 [Source:HGNC Symbol;Acc:HGNC:2240] | 4261 | 0.131 | 0.3829 | No |
| 15 | UBE2M | ubiquitin conjugating enzyme E2 M [Source:HGNC Symbol;Acc:HGNC:12491] | 4535 | 0.110 | 0.3765 | No |
| 16 | FN3K | fructosamine 3 kinase [Source:HGNC Symbol;Acc:HGNC:24822] | 4576 | 0.107 | 0.3877 | No |
| 17 | STT3A | STT3 oligosaccharyltransferase complex catalytic subunit A [Source:HGNC Symbol;Acc:HGNC:6172] | 4618 | 0.103 | 0.3984 | No |
| 18 | GPLD1 | glycosylphosphatidylinositol specific phospholipase D1 [Source:HGNC Symbol;Acc:HGNC:4459] | 5103 | 0.064 | 0.3694 | No |
| 19 | ABCA2 | ATP binding cassette subfamily A member 2 [Source:HGNC Symbol;Acc:HGNC:32] | 5349 | 0.044 | 0.3562 | No |
| 20 | ATG7 | autophagy related 7 [Source:HGNC Symbol;Acc:HGNC:16935] | 5488 | 0.033 | 0.3500 | No |
| 21 | CRTAP | cartilage associated protein [Source:HGNC Symbol;Acc:HGNC:2379] | 6700 | -0.055 | 0.2633 | No |
| 22 | UBA3 | ubiquitin like modifier activating enzyme 3 [Source:HGNC Symbol;Acc:HGNC:12470] | 6936 | -0.075 | 0.2551 | No |
| 23 | FOLH1 | folate hydrolase 1 [Source:HGNC Symbol;Acc:HGNC:3788] | 7048 | -0.083 | 0.2576 | No |
| 24 | FLCN | folliculin [Source:HGNC Symbol;Acc:HGNC:27310] | 8848 | -0.221 | 0.1475 | No |
| 25 | ATG12 | autophagy related 12 [Source:HGNC Symbol;Acc:HGNC:588] | 9776 | -0.299 | 0.1155 | No |
| 26 | AGTPBP1 | ATP/GTP binding carboxypeptidase 1 [Source:HGNC Symbol;Acc:HGNC:17258] | 10752 | -0.399 | 0.0932 | No |
| 27 | ATG16L1 | autophagy related 16 like 1 [Source:HGNC Symbol;Acc:HGNC:21498] | 11799 | -0.549 | 0.0855 | No |
Table: GSEA details [plain text format]

  

Fig 2: GOBP\_POST\_TRANSLATIONAL\_PROTEIN\_MODIFICATION      
 Blue-Pink O' Gram in the Space of the Analyzed GeneSet

  

Fig 3: GOBP\_POST\_TRANSLATIONAL\_PROTEIN\_MODIFICATION: Random ES distribution      
 Gene set null distribution of ES for **GOBP\_POST\_TRANSLATIONAL\_PROTEIN\_MODIFICATION**
